# A Markerless CRISPR-Mediated System for Genome Editing in Candida auris Reveals a Conserved Role for Cas5 in the Caspofungin Response

**DOI:** 10.1128/Spectrum.01820-21

**Published:** 2021-11-03

**Authors:** Craig L. Ennis, Aaron D. Hernday, Clarissa J. Nobile

**Affiliations:** a Department of Molecular and Cell Biology, School of Natural Sciences, University of California, Merced, California, USA; b Quantitative and Systems Biology Graduate Program, University of California, Merced, California, USA; c Health Sciences Research Institute, University of California, Merced, California, USA; The Ohio State University

**Keywords:** CRISPR, *Candida auris*, Cas5, Cas9, caspofungin, drug resistance mechanisms, genome editing, multidrug resistance

## Abstract

Candida auris is a multidrug-resistant human fungal pathogen that has recently emerged worldwide. It can cause life-threatening disseminated infections in humans, with mortality rates upwards of 50%. The molecular mechanisms underlying its multidrug resistance and pathogenic properties are largely unknown. Few methods exist for genome editing in C. auris, all of which rely on selectable markers that limit the number of modifications that can be made. Here, we present a markerless CRISPR/Cas9-mediated genome editing system in C. auris. Using this system, we successfully deleted genes of interest and subsequently reconstituted them at their native loci in isolates across all five C. auris clades. This system also enabled us to introduce precision genome edits to create translational fusions and single point mutations. Using Cas5 as a test case for this system, we discovered a conserved role for Cas5 in the caspofungin response between Candida albicans and C. auris. Overall, the development of a system for precise and facile genome editing in C. auris that can allow edits to be made in a high-throughput manner is a major step forward in improving our understanding of this important human fungal pathogen.

**IMPORTANCE** Candida auris is a recently emerged multidrug-resistant fungal pathogen capable of causing life-threatening systemic infections in humans. Few tools are available for genome editing in C. auris. Here, we present a markerless genome editing system for C. auris that relies on CRISPR/Cas9 technology and works to modify the genomes of all known C. auris clades. Using this system, we discovered a conserved role for Cas5 in the caspofungin response between C. albicans and C. auris. Overall, the development of a system for facile genome editing in C. auris is a major step forward in improving our understanding of this important human fungal pathogen.

## INTRODUCTION

Candida auris is a human fungal pathogen that can cause both superficial skin and mucosal infections, as well as disseminated bloodstream infections (1, 2). C. auris was first identified in Japan in 2009 from a patient with an external ear canal infection (3). Since then, C. auris has emerged worldwide, nearly simultaneously, across five different continents (4). Isolates of C. auris currently belong to five distinct geographic clades according to where they were first isolated: South Asia (clade I), East Asia (clade II), South Africa (clade III), South America (clade IV), and Iran (clade V) (4, 5). Based on current C. auris genome annotations, thousands of unique single nucleotide polymorphisms (SNPs) exist between the different clades (4–6). The majority of C. auris clinical isolates are resistant to one or more of the three major antifungal drug classes used to treat invasive infections (the azoles, echinocandins, and polyenes), nearly a third of isolates are resistant to two drug classes, and a few isolates are panresistant to all three of the major drug classes (4, 7). Within hospital settings, C. auris has been the cause of several outbreaks to date (7–10), and in the current coronavirus disease 2019 (COVID-19) pandemic, coinfections of C. auris with severe acute respiratory syndrome coronavirus 2 (SARS-CoV-2) have been increasingly reported (10–13). The mortality rates associated with C. auris infections are high (upwards of 50%) and are frequently attributed to treatment failures (14, 15). With its recent emergence and multidrug-resistant properties, C. auris is often referred to as a “superbug” that is a serious public health threat.

The ability to manipulate a microbial genome and identify genes and pathways of interest is a valuable tool in microbial genetics. In the *Candida* species, genome editing has historically been time and resource intensive, relying heavily on the incorporation of auxotrophic and/or resistance markers (16). Since its first application in Candida albicans in 2015 (17), CRISPR/Cas9-mediated genome editing has greatly increased the speed and efficiency of creating mutations in several *Candida* species (reviewed in reference 18). These systems rely on a unique RNA-guided DNA nuclease that directs a double-strand break (DSB) at a locus of interest. The DSB can be repaired by error-prone nonhomologous end joining (NHEJ) or by homology-directed repair (HDR), the latter relying on user-supplied repair templates to make mutations as small as a single base pair in size or to incorporate large heterologous sequences.

A few recent studies have created genome modifications in C. auris. Grahl et al. used a Cas9/RNA complex to create a DSB in the C. auris ortholog of *CAT1* in C. albicans, replacing the gene with the nourseothricin resistance marker gene (*NAT1*) (19). Similar methods have been utilized by two other groups to construct deletion mutant strains and to replace native promoters with inducible promoters (20, 21). Two studies utilized different selectable markers, such as nourseothricin and hygromycin B, to create gene deletion mutant strains in C. auris (21, 22). Collectively, these studies have developed and validated tools that have undoubtedly increased the pace of research on this important human fungal pathogen. However, all prior systems for genome editing in C. auris have relied on the incorporation of permanent selectable markers, ultimately reducing the numbers and types of genome modifications that are possible in a given strain. Additionally, the integration of markers and gene deletion cassettes has been demonstrated to be highly variable across C. auris clades (23), and all prior studies have genetically manipulated C. auris using clade I isolates only. Together, these issues highlight the need for the development of a facile and efficient genome editing system in C. auris that does not rely on the integration of permanent selectable markers and that is compatible with isolates across all known C. auris clades.

Here, we report a markerless CRISPR/Cas9-mediated genome editing system for C. auris, adapted from the C. albicans LEUpOUT system (24). This system relies on the temporary integration of Cas9 and target-specific guide RNA (gRNA) expression constructs by HDR at the C. auris *LEU2* locus and integration of a DNA repair template (“donor DNA,” or dDNA) at the target site of Cas9 cutting during transformations. After verification of the intended genome edits at the Cas9/gRNA target site, the gRNA, Cas9, and selectable marker expression cassettes are “recycled” and removed from the genome, resulting in a modified strain without the introduction of permanent markers. With this C. auris system, we successfully deleted and reconstituted the putative ortholog of C. albicans *CAS5* in a representative isolate from each of the five C. auris clades. *CAS5* encodes an important cell wall regulator in C. albicans that controls sensitivity to the echinocandin drug caspofungin, and we discovered that this function is conserved in C. auris. We further tested the versatility of our system by engineering a single point mutation at a conserved phosphorylation site in Cas5 and confirmed that this mutation prevents nuclear localization upon caspofungin treatment. Overall, this new C. auris CRISPR system enables rapid genome editing in C. auris to easily create deletion mutant strains and reconstituted (complementation/add-back) strains, as well as small-scale precision genome edits, which should significantly advance the field in understanding this important and recently emerged human fungal pathogen.

## RESULTS

### Construction of a markerless Candida auris CRISPR system.

**FIG 1:**
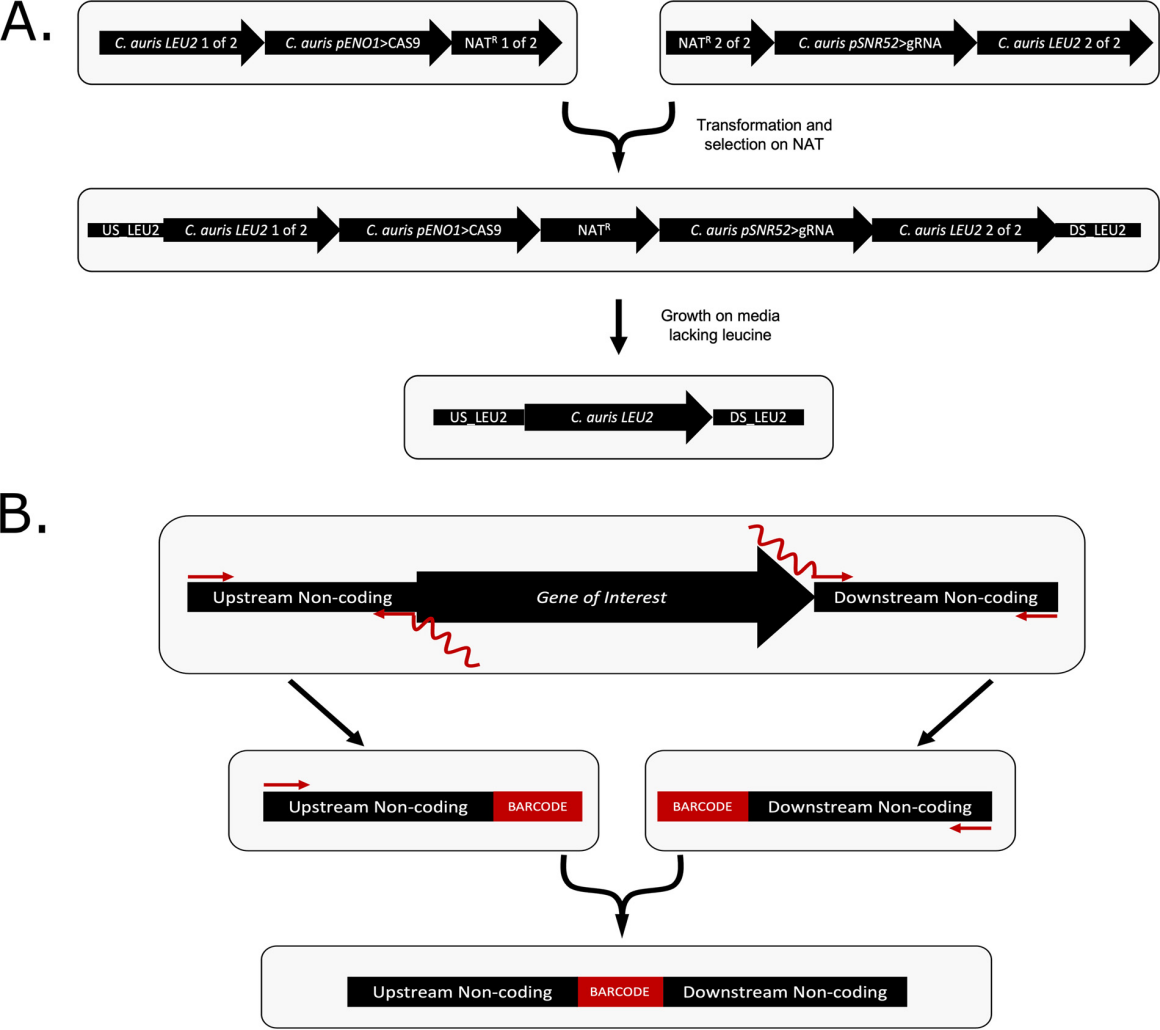
Schematic of the C. auris CRISPR system design. (A) Diagram of the individual and combined gRNA and Cas9 fragments and the transient C. auris genotypes at each stage of the transformation and LEUpOUT process. (B) Illustration of the repair template design used for constructing gene deletion strains. Two fragments (100 to 200 bp in length) are amplified and PCR stitched together to create a larger fragment (200 to 400 bp in length) with homology to the genome lacking the gene of interest.

The C. auris CRISPR system presented here is based on the widely used LEUpOUT system previously developed for C. albicans (24). Briefly, this system utilizes Cas9 and gRNA expression cassettes that are either amplified or digested from plasmids and then integrated within the *LEU2* locus to create a *LEU2* disruption strain that is resistant to nourseothricin or hygromycin B and auxotrophic for leucine (Fig. 1A). In contrast to the predominantly diploid strains of C. albicans, the majority of C. auris strains are haploid, and thus, the creation of a *LEU2/leu2Δ* hemizygous base strain is not typically required for genome edits in C. auris. Following confirmation of the intended edits at the target locus, which is determined by the unique gRNA sequence contained within the gRNA cassette, the CRISPR components are removed along with the resistance marker via selection on medium lacking leucine, which creates a selective pressure for cells that have undergone homologous recombination between the flanking direct repeats of the disrupted *LEU2* locus (Fig. 1A). This method for transient integration of the CRISPR components and the selectable marker enables efficient precision genome editing at the gRNA target locus and rapid marker recycling to allow essentially limitless serial genome editing.

The Cas9 plasmid contains homology to the 5′ end of the C. auris *LEU2* locus (B9J08_000229), with the C. auris *ENO1* promoter (B9J08_000274) driving the expression of Cas9 (Fig. 1A). This fragment contains part “1 of 2” of the nourseothricin gene (*NAT*) or hygromycin B gene (*HYG*) resistance selectable markers, while part “2 of 2” of the selectable markers is included in a separate gRNA plasmid. The “1 of 2” and “2 of 2” segments of each marker overlap by approximately 200 bp of homology, and the intact marker is reconstituted via homologous recombination following transformation into C. auris. In addition to the *NAT* or *HYG* 2-of-2 selectable markers, the gRNA construct carries the C. auris *SNR52* promoter, driving the expression of an *ADE2* (B9J08_003951) spacer sequence, which directs Cas9 to introduce DSBs at the target locus, fused to the conserved transactivating CRISPR RNA (tracrRNA) sequence that associates with Cas9 to direct cutting to the *ADE2* locus. This construct also contains homology to the 3′ end of *LEU2*, allowing these fragments to integrate at this locus.

### High-throughput gRNA design and amplification.

Our C. auris system enables simple, versatile, and high-throughput generation of unique gRNA expression cassettes via either of two simple PCR-mediated methods (24). The first method, the cloning-free method, involves PCR-based stitching of “universal A” and “unique B” fragments into a custom gRNA expression cassette that can be transformed directly into C. auris along with the accompanying Cas9 expression cassette. Using this method, a PCR plate full of unique gRNAs can be amplified in two reactions, followed by a PCR stitching step, enabling the quick creation of transformant libraries. Our gRNA design is described in detail in Text S1 in the supplemental material (“Candida auris Markerless CRISPR/Cas9 Genome Editing Protocol”), a protocol in which Data Set S1 (“Oligo Calculator”) was used to create a 60-bp oligonucleotide with standardized 20-bp sequences flanking the ends of the user-defined target sequence. The standardized flanking sequences facilitate the amplification of unique gRNAs using a standard PCR protocol. The second method, the cloning method, uses a single-oligonucleotide-based PCR strategy to generate unique gRNA expression cassettes via circularization of a linearized plasmid, followed by transformation into E. coli. The custom gRNA expression cassette can subsequently be excised from the cloned plasmid via restriction digestion and transformed directly into C. auris along with the accompanying Cas9 expression cassette. In either approach, only a single custom oligonucleotide is required to introduce a unique target sequence into the gRNA expression cassette, and this oligonucleotide can be designed using the “Oligo Calculator” in Data Set S1. Our base gRNA expression cassette, which is contained in plasmids pCE27 (containing the *NAT* resistance marker) and pCE41 (containing the *HYG* resistance marker), includes a spacer sequence that targets the C. auris *ADE2* locus, allowing users to easily perform *ADE2* editing as a control for CRISPR-mediated editing efficiency in C. auris. We note that the editing efficiency of our system at the C. auris *ADE2* locus is ∼20%. pCE27 and pCE41 also serve as templates for the incorporation of custom gRNA target sequences by either of the two methods mentioned above.

### Rapid generation of gene deletion strains across all C. auris clades.

**FIG 2:**
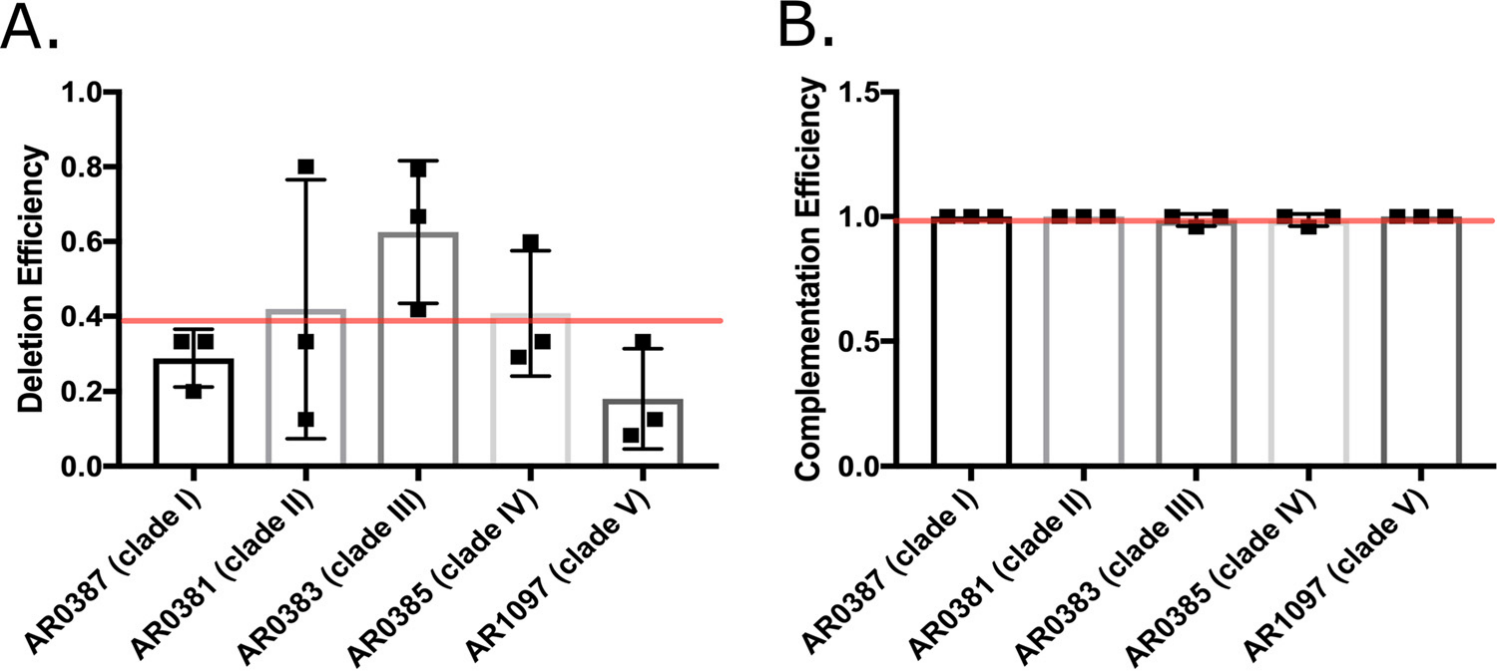
Editing efficiencies of *CAS5* deletion and complementation across C. auris clades. (A) *CAS5* was deleted in a representative isolate from each of the five C. auris genetic clades (AR0387 [clade I], AR0381 [clade 2], AR0383 [clade III], AR0385 [clade IV], and AR1097 [clade V]). All colonies obtained from each transformation were assessed. Three independent transformations were performed for each isolate. Average deletion efficiencies and standard deviations for each isolate were calculated. (B) *CAS5* was complemented to the native locus in a *cas5*Δ mutant from each genetic clade. All colonies obtained from each experiment were assessed. Three independent transformations were performed for each mutant strain. The average efficiency of *CAS5* integration and standard deviation for each strain constructed are shown.

A prior study has indicated that strains within C. auris clades III and IV have highly variable efficiencies for integration of selectable markers via homologous recombination (23); thus, we sought to determine whether our system could be effective for use in all C. auris clades. Using a representative isolate from each C. auris clade (AR0387 [clade I], AR0381 [clade II], AR0383 [clade III], AR0385 [clade IV], and AR1097 [clade V]), we measured editing efficiency by deleting the C. auris gene (B9J08_000765) that is a putative ortholog of the C. albicans *CAS5* gene. These C. auris base strains were selected because they have been previously used in virulence studies and have predetermined MICs for the three major antifungal drug classes used in the clinic. In C. albicans, *CAS5* encodes the extensively studied transcription factor Cas5 that controls cell wall integrity, and C. albicans *cas5*Δ/Δ mutants exhibit hypersensitivity to the echinocandin drug caspofungin (25–27). C. auris transformations were performed on three separate occasions using the *NAT* selectable marker version of the LEUpOUT system (Fig. 1A) along with amplified dDNA with a 20-bp barcode sequence (Fig. 1B and described below), and nourseothricin-resistant colonies were screened for deletion of *CAS5* by PCR genotyping. Transformations into the representative strain from clade I (AR0387) revealed *CAS5* deletion efficiencies similar to the across-clade average of 40% (Fig. 2A). We observed the highest efficiency (62%) of *CAS5* deletion in the representative clade III isolate (AR0383), while the clade V isolate (AR1097) exhibited the lowest efficiency (18%). Nonetheless, we were successful in obtaining *cas5*Δ strains from each of our representative clinical isolates with good efficiency. Overall, these results demonstrate our ability to construct markerless deletion mutant strains in C. auris.

We note that while developing this method, we tested various approaches for generating the necessary dDNA fragments that result in the deletion of a target gene upon integration via homologous recombination at the site of Cas9 cutting. We first attempted to create gene deletion mutants using relatively short dDNA fragments with 50 to 70 bp of homology to sequences on either side of the Cas9/gRNA target locus, which is typically sufficient for comparable C. albicans transformations; however, we observed extremely low editing efficiencies using these shorter fragments. To overcome this issue, we utilized a PCR stitching approach to generate dDNA fragments with 100 to 200 bp of upstream and downstream flanking homology to the C. auris *CAS5* (B9J08_000765) open reading frame (ORF), using primers that bridge the upstream and downstream fragments together via a 22-bp barcode (Fig. 1B). Since the barcode sequence is not found within the C. auris genome and contains an additional GG sequence, it also serves as a unique gRNA target sequence for subsequent Cas9-mediated editing of the deleted locus.

We also note that during PCR-based screening for *CAS5* deletion transformants, we identified a low frequency (2 to 6%) of colonies across isolates that had duplicated the *CAS5* locus (duplication of *CAS5* was most frequent in the clade V isolate, at a frequency of ∼6%). These duplication events were observed while using either the *NAT* or *HYG* selectable markers and were evident based on the presence of distinct PCR bands signifying one intact copy and one deleted copy of *CAS5*. Both PCR bands were observed after obtaining single-colony isolates, confirming that these distinct bands were not the result of background growth of wild-type cells on the transformation plate.

### Facile complementation of deleted genes at their native loci.

Since the dDNA used to delete *CAS5* (Fig. 1B) created a unique “landing pad” site for subsequent genome editing, we tested the ability of this system to complement (i.e., add back) the deleted *CAS5* gene at its native locus. Similar landing pads, also called “AddTags,” have been previously utilized in C. albicans to reintegrate deleted genes at their native loci (24, 28). Briefly, we generated a new gRNA cassette to target our landing pad using the *HYG* selectable marker version of the C. auris LEUpOUT system. We then amplified our *CAS5* add-back dDNA via PCR amplification of C. auris genomic DNA from a wild-type C. auris strain using primers that flank the *CAS5* gene to generate 80 bp of upstream and downstream flanking homology to the *cas5*Δ landing pad. Using this method, we were successful in complementing the strains from each of the five clades with the *HYG* selectable marker, noting exceptionally high overall efficiency (99%) (Fig. 2B). Together, these results indicate that our *HYG* and/or *NAT* selectable marker constructs support facile deletion and complementation of genes in representative isolates from each of the five C. auris clades.

### Cas5 is a conserved regulator of the caspofungin response in C. auris.

**FIG 3:**
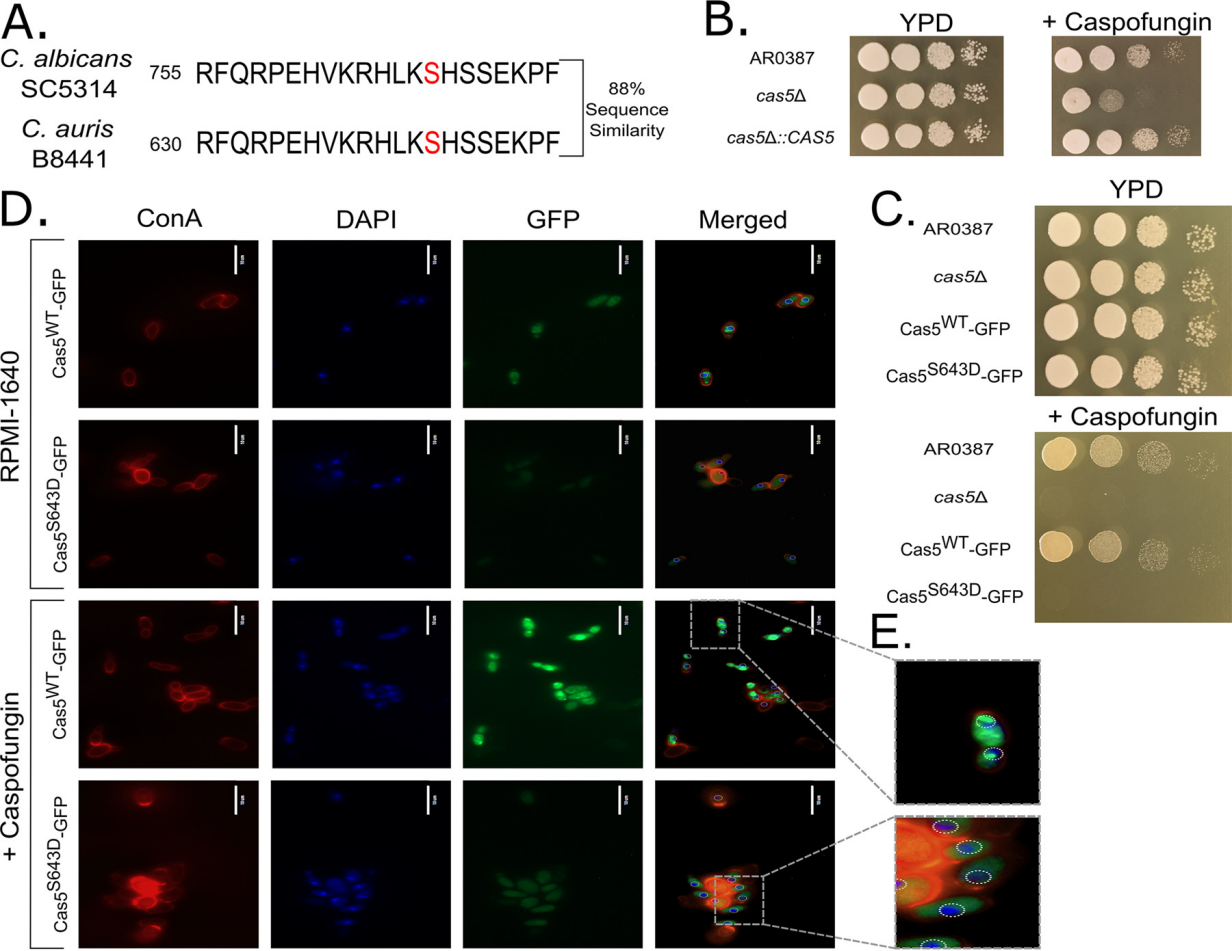
Cas5 is a conserved regulator of the caspofungin response. (A) Alignment of the C. albicans and C. auris Cas5 amino acid sequences using BLASTP, focusing on a portion of the conserved DNA-binding domain and phosphorylation site (red). (B) Serial spot dilution plate assays performed on YPD medium and YPD medium supplemented with 62.5 ng/ml caspofungin with the AR0387 (clade I) wild-type, *cas5*Δ, and *cas5*Δ::*CAS5* strains. Plates were grown at 30°C, and images were acquired after 24 h of growth. (C) Serial spot dilution plate assays performed on YPD medium and YPD medium supplemented with 62.5 ng/ml caspofungin with the AR0387 (clade I) wild-type, *cas5*Δ, Cas5^WT^-GFP, and Cas5^S643D^-GFP strains. Plates were grown at 30°C, and images were acquired after 24 h of growth. (D) Fluorescence microscopy images of the Cas5^WT^-GFP and Cas5^S643D^-GFP strains incubated in the presence and absence of 125 ng/ml caspofungin. Fungal cell walls were stained with ConA, and nuclei were stained with DAPI. Cells were imaged with a 100× oil immersion objective. Scale bars represent 10 μm. (E) Representative zoomed-in inset images from a subset of representative cells of the Cas5^WT^-GFP and Cas5^S643D^-GFP strains in panel D, depicting GFP localization after incubation with 125 ng/ml caspofungin.

Nearly half of the predicted proteins encoded within the C. auris genome have putative orthologs in C. albicans (6). One of many putative orthologs that remains uncharacterized is the transcriptional regulator Cas5. In C. albicans, *CAS5* encodes a well-characterized transcriptional regulator of cell wall integrity, and the deletion of *CAS5* results in hypersensitivity to the frequently used antifungal drug caspofungin (25–27). A global alignment between C. albicans Cas5 and its putative ortholog in C. auris (B9J08_000765) using BLASTP revealed 88% conservation of the DNA-binding domain (C. albicans, positions 743 to 801, and C. auris, positions 617 to 675) (Fig. 3A) and 33% conservation across the entire protein; thus, we asked whether this putative Cas5 ortholog retains the same function in C. auris. Caspofungin sensitivity analyses of the C. auris clade I wild-type (WT) strain and its corresponding *cas5*Δ mutant and complementation strains by plate spot assays and growth rate experiments confirmed that Cas5 is indeed a conserved regulator of the caspofungin response in C. auris (Fig. 3B and Fig. S1).

### Efficient green fluorescent protein (GFP) tagging and codon editing in C. auris.

In general, the C. auris genome is largely unexplored, and many of its genes remain uncharacterized. Using Cas5 as our test case, we also asked whether our C. auris genome editing system could support the integration of heterologous sequences, such as fluorescent protein coding sequences, and the introduction of small-scale edits, such as codon substitutions. C. albicans Cas5 is localized to the cytoplasm under basal conditions and is localized in the nucleus during caspofungin treatment (26), and this translocation is controlled by the dephosphorylation of a serine residue at amino acid position 769. The C-terminal DNA-binding domain, as well as this putative phosphorylation site (S643), is highly conserved between the two species (Fig. 3A). We first created a Cas5-GFP C-terminal translational fusion (100% editing efficiency) using a CTG-optimized enhanced GFP (eGFP) coding sequence that also encodes a 7-amino-acid linker sequence (RIPLING). Cas9 was directed to induce DSBs at a site 10 bp upstream from the *CAS5* stop codon, and the dDNA used to introduce GFP was designed to ablate this Cas9 target site upon successful integration at the *CAS5* locus, to create a Cas5^WT^-GFP C. auris strain. We note that the dDNA for this purpose utilized 52 bp of homology, which is substantially less homology than was required for making gene deletion strains. Plate spot assays and fluorescence microscopy confirmed the functionality of our Cas5^WT^-GFP fusion C. auris strain and that C. auris Cas5 also translocates from the cytoplasm to the nucleus upon exposure to caspofungin (Fig. 3C to E).

We next asked whether we could use our genome editing system to mutate the conserved serine residue in our GFP-tagged C. auris strains to create an amino acid substitution (S643D) that should mimic a constitutively phosphorylated Cas5. We directed Cas9 to cut at a target sequence that overlaps the S643 codon (AGC) and used the same dDNA stitching approach outlined in Fig. 1B to introduce an S643D substitution (GAT) into a C. auris strain that already harbored our Cas5-GFP translational fusion. Since this codon partially overlaps the reverse complement of the TGG protospacer adjacent motif (PAM) site used to target Cas9 cutting at this location, the incorporation of our modified dDNA ablated this PAM site and replaced it with the sequence TGA. To further ensure that the dDNA or modified target site would not be cut by Cas9, eight synonymous substitutions were also incorporated within the region of the dDNA representing the original 20-bp gRNA target site (40% of the sequence altered), while retaining the wild-type amino acid-coding sequence (10% editing efficiency). This newly constructed Cas5^S643D^-GFP C. auris strain was then assessed for GFP localization and caspofungin sensitivity. As expected, the S643D substitution caused C. auris Cas5 to largely remain in the cytoplasm, even in the presence of caspofungin (Fig. 3D and E). Quantitation of the Cas5 cellular localization results from the microscopy images for each of the strains tested was as follows: Cas5^WT^-GFP (untreated) = 80% ± 10% cytoplasmic (mean ± standard deviation) and 20% ± 10% nuclear; Cas5^WT^-GFP (caspofungin treated) = 31% ± 10% cytoplasmic and 69% ± 10% nuclear; Cas5^S643D^-GFP (untreated) = 100% ± 0% cytoplasmic and 0% ± 1% nuclear; and Cas5^S643D^-GFP (caspofungin treated) = 76% ± 6% cytoplasmic and 24% ± 6% nuclear. Furthermore, caspofungin sensitivity analysis confirmed that the Cas5^S643D^-GFP C. auris strain is hypersensitive to caspofungin (Fig. 3C to E), which is consistent with previously published results for C. albicans (26). Taken together, these results highlight not only the conserved function and regulation of Cas5 between C. auris and C. albicans but also the utility of our C. auris genome editing system to incorporate heterologous sequences and explore the consequences of small-scale precision genome edits on gene function.

## DISCUSSION

The recent emergence of C. auris, coupled with its high virulence and inherent antifungal drug resistant properties, make it a pressing microorganism for study. Here, we developed a genome editing system that enables efficient, facile, and inexpensive precision genome editing in C. auris. An additional advantage of this system is that it does not rely on the incorporation of permanent selectable markers, and the Cas9 and gRNA expression cassettes are removed along with the selectable marker following successful verification of the intended genome edits at the Cas9 target site. In this way, the system enables essentially limitless serial genome edits in the same genetic background. We display the versatility of our system to make sequential edits in clinical isolates in two different ways. First, gene deletion strains of our gene of interest in all five C. auris clades were made that were subsequently complemented at their native loci. Second, we created a translational fusion with GFP followed by an additional edit to introduce a nonsynonymous substitution within the coding sequence of the GFP-tagged gene of interest.

Since most C. auris clinical isolates are haploid and, thus, directly compatible with our LEUpOUT system, most C. auris isolates should be amenable to our genome editing system without requiring any prior strain engineering. One group has recently identified highly virulent diploid isolates of C. auris (29), which would require prior strain modification to be compatible with our genome editing system; however, we anticipate that the generation of *LEU2* hemizygous strains using traditional gene deletion approaches should enable the use of our genome editing system in C. auris diploids, as has been demonstrated with a similar genome editing system in C. albicans (24).

Two sets of plasmids are available for use in our genome editing system, each relying on a different selectable marker, allowing transformation into strains previously constructed with a selectable marker. For example, if one were to attempt genome editing in a diploid C. auris isolate, it should be possible to use the *NAT* marker system to delete a single copy of the *LEU2* gene via integrative transformation and then subsequently use the *HYG* selectable marker system for precision genome editing. Beyond the versatility of having two selectable markers, the *HYG* selectable marker system reduces the cost of transforming strains, since hygromycin B is a more affordable alternative to nourseothricin. In our experience, the *HYG* selectable marker system is superior to the *NAT* selectable marker system at reducing background growth of C. auris on selective medium. This variable background growth can make isolating single colonies more difficult if performing a subsequent transformation with the *NAT* marker system. While both systems work well for markerless genome editing in C. auris, we find that using hygromycin B provides a more robust selection with consistently lower background colony growth than using nourseothricin, making subsequent complementation and downstream edits easier.

While the genome editing system presented here is based on the widely used C. albicans LEUpOUT system published by Nguyen et al. (24), the plasmids and repair template designed here were modified and optimized to increase the editing efficiency in C. auris. When developing this system, we initially replaced the C. albicans *LEU2* with the C. auris *LEU2* sequence, while retaining the native C. albicans promoters, to test whether these heterologous promoters were sufficient for gene editing. With this plasmid design, C. auris readily incorporated the CRISPR components, yet *cas5*Δ mutants were exceptionally rare in all isolates tested. With the additional incorporation of C. auris-specific promoters (*SNR52* and *ENO1*), we were able to successfully perform genome editing at the *CAS5* locus across all clades and isolates tested.

We tested several different repair template designs to further improve efficiency. Previous studies have used repair templates with as little as 50 bp homology upstream and downstream from the target locus (22, 23). Using annealed oligonucleotides with 40 to 50 bp homology, as is common with the C. albicans system, gene deletion mutants with the new plasmids were infrequent in clades I and IV and were exceptionally rare in clades II, III, and IV, whereas increasing the homology length to 100 to 200 bp substantially increased our gene deletion efficiencies (across-clade average of 40% for deleting *CAS5*). Interestingly, the repair template used for creating the Cas5-GFP translational fusion strain contained 52 bp homology upstream and downstream from the target locus and was more than sufficient for GFP tagging (100% efficiency), indicating that C. auris can integrate sequences that contain shorter regions of homology with high efficiency. Furthermore, we observed extremely high (99%) complementation efficiency for adding back *CAS5* to its native locus. The C. albicans LEUpOUT system boasts editing efficiencies for complementation ranging from 60 to 80% (24), requiring the integration of two copies of the gene of interest in a diploid organism. We believe that the higher complementation efficiency observed here is due to the haploid genome architecture of C. auris, requiring the integration of only a single copy of the gene of interest. In addition to deleting and complementing entire protein coding genes, we also demonstrate the ability to modify single codons as a proof of concept that this system can also be used to study the functional consequences of small-scale genome edits.

Genomic rearrangements and aneuploidy are common stress response mechanisms in the *Candida* species (30, 31), and C. auris is no exception. Genomic rearrangements are frequent across C. auris isolates and clades, with the highest degree of rearrangements and gene losses occurring in clade II isolates (32, 33). While the mechanism of the *CAS5* duplication events observed in our deletion transformations is unknown, we note that the standard lithium acetate fungal transformation process is known to induce genomic rearrangements and aneuploidy in C. albicans (34). A common concern regarding Cas9-mediated genome editing is the potential for off-target cutting, which could result in unwanted genetic modifications; however, we do not believe this is the primary mechanism responsible for the *CAS5* duplication events that we observed. Two independent studies have concluded that off-target effects of Cas9 genome editing in C. albicans are likely to be rare (34, 35); however, Marton et al. found that lithium acetate-based transformations frequently induce genomic rearrangements, regardless of whether or not Cas9 is used in the method (34). Furthermore, off-target cutting is believed to be more of a concern in larger eukaryotic genomes (36), such as those of mammals, which have ∼500-fold larger genomes than *Candida* species and thus contain fewer unique 20-bp sequences from which to select gRNA targets. Even so, off-target cutting in mammalian genomes has been shown to be relatively rare, even at sites that differ from the intended target by as few as three nucleotides (37, 38). Considering the relatively small genome size of C. auris and assuming the selection of highly specific 20-bp gRNA target sites, we believe that off-target cutting is unlikely to be a significant problem when using our genome editing system. However, the observation of *CAS5* duplication events highlights the importance of careful evaluation of engineered strains (through, for example, genetic complementation experiments), regardless of the transformation methodology being used. In addition, our facile gene complementation approach is another advantage of our system that can enable researchers to easily control and monitor for unintended modifications in gene deletion strains.

While assessing the functionality of our genome editing system, we chose to focus on *CAS5* in C. auris because its ortholog in C. albicans is well characterized and its deletion in C. albicans results in a clearly discernible hypersensitivity to caspofungin. Alignment of C. albicans and C. auris Cas5 sequences indicates that the C-terminal domain containing the DNA-binding domain and phosphorylation site is highly conserved (88% identity), while the remaining amino acid sequences have diverged greatly (33% identity). Based on this striking sequence identity within the DNA-binding domain, we hypothesized that the function of Cas5 is likely to be conserved between the two species, despite the fact that the two species are evolutionarily quite distant. Using this system, we constructed a GFP-tagged Cas5 C. auris strain containing codon substitutions that mimic a constitutively phosphorylated form of Cas5 (a Cas5^S643D^-GFP strain), and we observed that this strain was hypersensitive to caspofungin, matching the phenotype of the *cas5*Δ strain. We observed decreased GFP fluorescence by microscopy in the Cas5^S643D^-GFP C. auris strain relative to the fluorescence in the Cas5^WT^-GFP C. auris strain. This finding could be the result of Cas5 regulating its own expression and would be consistent with previous work in C. albicans that observed decreased polymerase II (Pol II) occupancy at the *CAS5* locus in the *cas5*Δ/Δ C. albicans strain compared to that in the wild-type C. albicans strain in both the presence and absence of caspofungin (26). Interestingly, we observed an increase in GFP fluorescence under caspofungin treatment in the Cas5^S643D^-GFP C. auris strain. We suspect that either the Cas5^S643D^-GFP C. auris strain is still capable of inducing its own expression (26) or there are unidentified transcriptional regulators contributing to caspofungin-induced *CAS5* expression in C. auris. Additionally, our findings suggest that the caspofungin hypersensitivity of this Cas5^S643D^-GFP C. auris strain is due to the inability of this modified form of Cas5 to effectively translocate into the nucleus in response to caspofungin treatment. Overall, consistent with its role in C. albicans, we discovered that Cas5 in C. auris is also a key regulator of the caspofungin response.

Broadly, this study contributes to our understanding of the cell wall stress response in *Candida* species. The CTG clade, which is named for species that translate CTG as serine rather than leucine (39), comprises many *Candida* species (e.g., C. albicans, Candida dubliniensis, Candida parapsilosis, and C. auris) and excludes certain *Candida* species (e.g., Candida glabrata and Candida krusei). The most recent common ancestor of C. albicans and S. cerevisiae diverged approximately 235 million years ago, with S. cerevisiae lacking a Cas5 ortholog (26, 40). Prior to this study, Cas5 was identified as a critical caspofungin response regulator in only C. albicans and C. parapsilosis (25, 41). The evolutionary distance between C. albicans and C. auris is large, much larger than the evolutionary distance between C. albicans and C. parapsilosis (6), and thus, the conserved role of Cas5 in the caspofungin response in C. auris that we observe here is not necessarily expected. In the non-CTG clade species C. glabrata, *MOT3* encodes a transcriptional regulator with synteny to both *CAS5* in C. albicans and *MOT3* in S. cerevisiae; however, Mot3 in C. glabrata is dispensable for the caspofungin response (42, 43). C. krusei has a “Cas5-like” regulator with greater conservation at the DNA-binding domain to Cas5 in C. albicans (66% identity) than to Mot3 in S. cerevisiae (48% identity) or Mot3 in C. glabrata (37% identity) (26), and yet, a role for this Cas5-like regulator in C. krusei has yet to be established. Future work characterizing putative Cas5 orthologs across the *Candida* species would greatly improve our understanding of how these important human fungal pathogens respond to cell wall stress.

Essential genes are promising drug targets in fungal pathogens, as interference with their protein products leads to fungal cell death (17, 18, 44). As such, essential genes are not amenable to deletion, and yet, their expression can be controlled using CRISPR/Cas9 technologies (18). A CRISPR interference (CRISPRi) system utilizing a catalytically inactive Cas9 (dCas9) for stable gene repression is available in C. albicans and has been demonstrated to be a useful tool for studying essential genes (45). A similar system using dCas9-tethered transcriptional activation domains has been shown to be an effective means of targeted transcriptional activation in C. albicans (46). Adaptation of our genome editing system to incorporate similar tools to allow for facile repression or activation of genes of interest in C. auris should facilitate the future identification of fungal-specific drug targets in C. auris.

In summary, we present a versatile CRISPR/Cas9-mediated genome editing system for use in C. auris that is the first system that does not create a terminal strain retaining a selectable marker in the genome. We believe this system is a major genetic advance in the field that should facilitate future discoveries in the biology of this important and recently emerged human fungal pathogen.

## MATERIALS AND METHODS

### Strains and culturing conditions.

Wild-type C. auris clinical isolate strains AR0387 (clade I), AR0381 (clade II), AR0383 (clade III), AR0385 (clade IV), and AR1097 (clade V) used in this study were obtained from the Centers for Disease Control and Prevention (CDC) and U.S. Food and Drug Administration (FDA) Antimicrobial Resistance (AR) Isolate Bank, Drug Resistance *Candida* species panel (https://wwwn.cdc.gov/ARIsolateBank/, accessed 24 May 2021). Transformed strains constructed in this study are listed in Data Set S2, and oligonucleotides used in this study are listed in Data Set S3. A detailed protocol describing necessary regents, dDNA and gRNA amplification procedures, and the C. auris transformation process is available in Text S1 (“Candida auris Markerless CRISPR/Cas9 Genome Editing Protocol”).

Genotypes of the edited strains were verified by colony PCR. The *cas5*Δ mutant strains (CEC97 [AR0383], CEC98 [AR0385], CEC99 [AR0387], CEC100 [AR1097], and CEC140 [AR0381]) were made with stitched PCR products from pCE27 and MssI-digested pCE35 in their respective isogenic wild-type strains. The complemented strains (CEC141 [AR0383], CEC142 [AR0385], CEC183 [AR0387], CEC144 [AR1097], and CEC165 [AR0381]) were made in their respective *cas5*Δ deletion mutant strains with stitched PCR products from pCE41 and MssI-digested pCE38. Wild-type *CAS5* for complemented strains was amplified from genomic DNA from each isolate with flanking homology to the *CAS5* upstream and downstream noncoding regions. The Cas5^WT^-GFP strain (CEC68) was made by amplifying *Candida* optimized monomeric GFP (pCE1) with a short linker (RIPLING) with flanking homology to the 3′ end of *CAS5* and the downstream noncoding region of the gene. This GFP-tagged strain was edited an additional time with dDNA amplified from the Cas5^WT^-GFP strain (CEC68) genomic DNA using primers CJNO4368, CJNO4369, CJNO4372, and CJNO4373 to introduce a mutation creating a Cas5^S643D^-GFP strain (CEC146), which was verified by PCR and Sanger sequencing. The oligonucleotides used to amplify the Cas5^S643D^ dDNA contained synonymous point mutations to ablate future Cas9 cutting and a nonsynonymous substitution to introduce the S643D mutation. Changes to the DNA sequence in the Cas5^S643D^-GFP strain are denoted with bold lowercase font in Data Set S3.

C. auris cells were stored at −80°C and were streaked on YPD plates (2% peptone, 2% dextrose, and 1% yeast extract) and grown for 2 days at 30°C. Single colonies were inoculated into liquid YPD medium and grown overnight with shaking at 30°C for use in all downstream experiments.

### Plasmid construction.

Plasmids pADH118 and pADH137 (24) were used as the base plasmids to construct the gRNA and Cas9 plasmids, respectively. All primers were designed based on the strain B8441 (AR0387) genome, and B8441 genomic DNA was used for amplification of all promoters. The gRNA plasmid was constructed by PCR amplification of pADH118 to integrate the 3′ end of the B8441 B9J08_000229 (*LEU2*) ORF, which was amplified using genomic DNA and gap repaired using chemically competent DH5α E. coli (catalog number 60107-1; Lucigen), creating pCE8. pCE8 was further linearized by PCR amplification to remove the C. albicans *SNR52* promoter, which was replaced with the C. auris *SNR52* promoter amplified using genomic DNA. This again was gap repaired with chemically competent DH5α E. coli to create pCE27. The Cas9 plasmid was constructed by linearizing pADH137 and the 5′ end of the of the B8441 B9J08_000229 (*LEU2*) ORF, which was amplified using genomic DNA and integrated using gap repair to create pCE4. This plasmid was linearized an additional time to replace the *ENO1* promoter driving the expression of Cas9 with gap repair to create pCE35.

The plasmid containing monomeric GFP was constructed by gap repair cloning into a linearized pADH98 entry vector created by PCR amplification followed by DpnI digestion and gel extraction. GFP was codon optimized for CUG clade species and purchased from ThermoFisher Scientific with homology to the pADH98 entry vector added to each end. This synthetic fragment and entry vector was gap repaired as described above using chemically competent DH5α E. coli to create pCE1. All plasmids constructed in this study were verified by sequencing and are available on AddGene with the following accession numbers (pCE1 [174434], pCE27 [174405], pCE35 [174409], pCE38 [174432], and pCE41 [174433]).

### Transformation of C. auris cells by heat shock.

C. auris cells were transformed as previously described for C. albicans cells (24). Briefly, C. auris overnight cultures were diluted to an optical density at 600 nm (OD_600_) of 0.1 in YPD medium and grown at 30°C shaking until they reached an OD_600_ between 0.4 to 0.7. Cells were pelleted and washed twice in sterile water before being transferred to a DNA mixture of the unique gRNA fragment, Cas9 fragment, dDNA repair template, and single-stranded salmon sperm DNA (preboiled). Fresh PLATE mix (50% polyethylene glycol 3350 [PEG-3350], 25 mM lithium acetate, 1× Tris-EDTA [TE]) was added to each transformation culture and incubated overnight. Incubated cells were heat shocked at 44°C for 15 min, washed, and recovered in YPD medium at 30°C with shaking for 4 h before plating on YPD medium supplemented with 200 μg/ml nourseothricin or 500 μg/ml hygromycin B. Individual colonies were checked after 2 days of growth at 30°C, patched onto YPD plates supplemented with 200 μg/ml nourseothricin or 500 μg/ml hygromycin B, and verified by colony PCR for the genome edit of interest. Colonies that had the correct genome edit were restreaked on synthetic complete (SC) medium lacking leucine and isoleucine (6.7% yeast nitrogen base without ammonium sulfate, 2% glucose, and auxotrophic supplements) to remove the CRISPR components. Individual colonies were patched onto YPD plates with and without 200 μg/ml nourseothricin or 500 μg/ml hygromycin B to confirm loss of the *NAT* or *HYG* resistance markers and CRISPR components.

### Plate spot dilution assays.

Overnight cell cultures were diluted to an OD_600_ of 0.2 in YPD medium and grown to mid-log phase with shaking at 30°C. The OD_600_ of the back-diluted culture was measured and diluted to an OD_600_ of 0.2. This was serially diluted 1:10 four times for each strain, and the dilutions plated onto YPD plates supplemented with and without 62.5 ng/ml caspofungin. Plates were incubated at 30°C for 24 h and imaged.

### Fluorescence microscopy.

C. auris Cas5^WT^-GFP and Cas5^S643D^-GFP strains (CEC68 and CEC146) were grown overnight, diluted to an OD_600_ of 0.5 in RPMI 1640 medium buffered with MOPS (morpholinepropanesulfonic acid), and incubated at 30°C in the presence and absence of 125 ng/ml caspofungin for 60 min. Cells were fixed with 2% formaldehyde, stained with 4′,6-diamidino-2-phenylindole (DAPI) and concanavalin A (ConA), and washed prior to being imaged on an EVOS FL microscope with a 100× oil immersion objective using the DAPI, GFP, and Texas red filters. Quantification of nuclear and cytoplasmic Cas5-GFP localization was determined by counting at least 100 cells from representative images of each strain using the Fiji software package (47) in ImageJ (48). The proportion of cells with nuclear or cytoplasmic Cas5 localization was averaged across all images.

### Growth curves.

Growth curves were performed by diluting overnight C. auris cultures to 10^5^ CFU/ml in RPMI 1640 medium buffered with MOPS. Cells were further diluted (3:4) in a 384-well plate with RPMI 1640 medium buffered with MOPS with or without caspofungin at a concentration of 62.5 ng/ml and grown for 32 h with linear shaking at 30°C in a BioTek Cytation 5 plate reader. The OD_600_ of each well was measured at 20-min intervals. The averages of 12 replicates per strain and treatment condition were calculated.

### Data availability.

Requests for resources and reagents should be directed to Clarissa Nobile (cnobile@ucmerced.edu). All plasmids constructed in this study are available on AddGene with the following accession numbers: pCE1, 174434; pCE27, 174405; pCE35, 174409; pCE38, 174432; pCE41, 174433.
