# Supplementary material for: A Markerless CRISPR-Mediated System for Genome Editing in Candida auris Reveals a Conserved Role for Cas5 in the Caspofungin Response
Source: Microbiol Spectr. 2021 Nov 3;9(3):e01820-21. doi: 10.1128/Spectrum.01820-21 (PMC8567271; doi:10.1128/Spectrum.01820-21)
Supplement: SUPPLEMENTAL FILE 1 — Supplemental material. Download SPECTRUM01820-21_Supp_1_seq6.pdf, PDF file, 0.9 MB [file spectrum01820-21_supp_1_seq6.pdf]

Candida auris Markerless CRISPR/Cas9 Genome Editing Protocol

Updated 07/01/2021

Reagents and Media

- Phusion™ High Fidelity Polymerase (Thermo Scientific™ F530L)
- FastDigest *Mss*I (Thermo Scientific™ FD1344)
- 10 mM dNTP
- 1 M Lithium Acetate
- 10X TE
- Sterile dH_2O
- Liquid YPD
- YPD plates supplemented with 300 mg/mL Nourseothricin (YPD + NAT300) or supplemented with 600 mg/mL HygromycinB (HYG600)
- LB supplemented with carbenicillin
- 50% PEG
- LB plates supplemented with 100 µg/mL carbenicillin
- SC with auxotrophic supplements lacking leucine
- Salmon sperm DNA

Consumables

- 1.5 mL tubes
- 50 mL conical tubes
- Mini or Midi Prep Kit
- 10 mL snap top tubes

Primers

Universal A Fragment Forward (AHO1096): GACGGCACGGCCACGCGTTTAAACCGCC
Universal A Fragment Reverse (CJNO3235): TGTTTTCTGCTGAGGGAGTC
Universal B Fragment Reverse (AHO1097): CCCGCCAGGCGCTGGGGTTTAAACACCG
Universal C Fragment Forward (NAT Marker) (AHO1237): AGGTGATGCTGAAGCTATTGAAG
Universal C Fragment Forward (HYG Marker) (CJNO3755): TACTGTATAAGTCGAAGAGCACAAG
Universal C Fragment Reverse (CJNO3080): TTATTTCTGCAAAAGCTTCTTTAC
Unique 60bp gRNA (see gRNA Design section for details)
Unique dDNA primers (four total)
PCR primers for checking transformations

Plasmids

All plasmids use a *bla* marker and should be cultured using LB supplemented with carbenicillin

- pCE27 – *C. auris* NAT gRNA Plasmid with *ADE2* gRNA
    - This plasmid contains the full length *ADE2* C Fragment for transformation and can be digested with *Mss*I or amplified with the C fragment primers and used as a control. Alternatively, Universal A and Unique B Fragments can be amplified with the above primers.
- pCE35 – *C. auris* NAT Cas9 Plasmid
    - This plasmid is to be digested with *Mss*I
- pCE41 - *C. auris* HYG gRNA Plasmid with *ADE2* gRNA

    - See information above for pCE27
- pCE38 – C. auris HYG Cas9 Plasmid
    - See information above for pCE35

---

gRNA Design

The *C. auris* B8441 has been uploaded to Benchling to easily identify gRNAs for transformation. Use the steps as follows to select gRNAs:

1. Upload your gene of interest with 1 kB upstream and downstream into Benchling
2. Select the region you wish to target with a gRNA

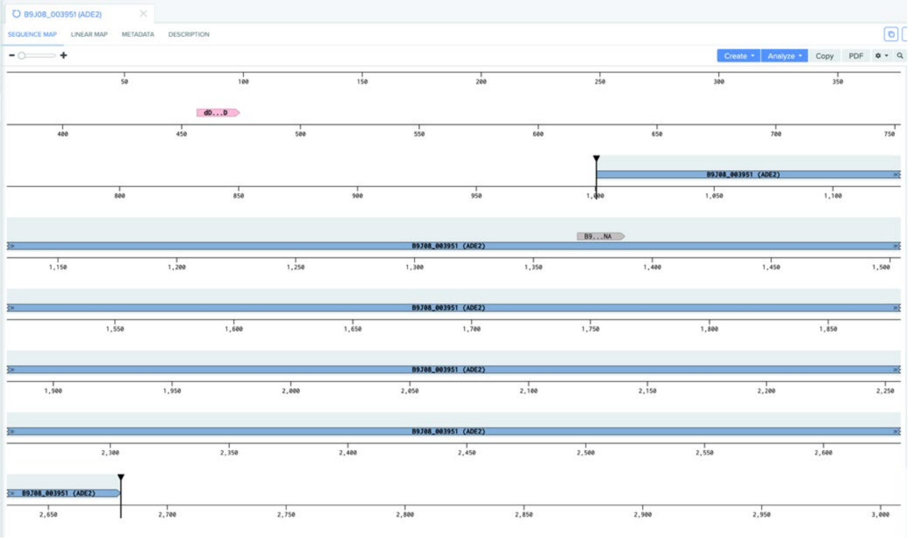

3. Use the gRNA selection tool located on the right side of the Benchling interface

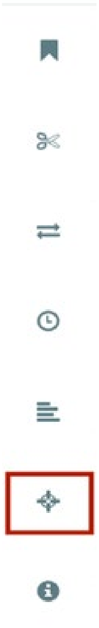

5. Set the guide parameters to the following

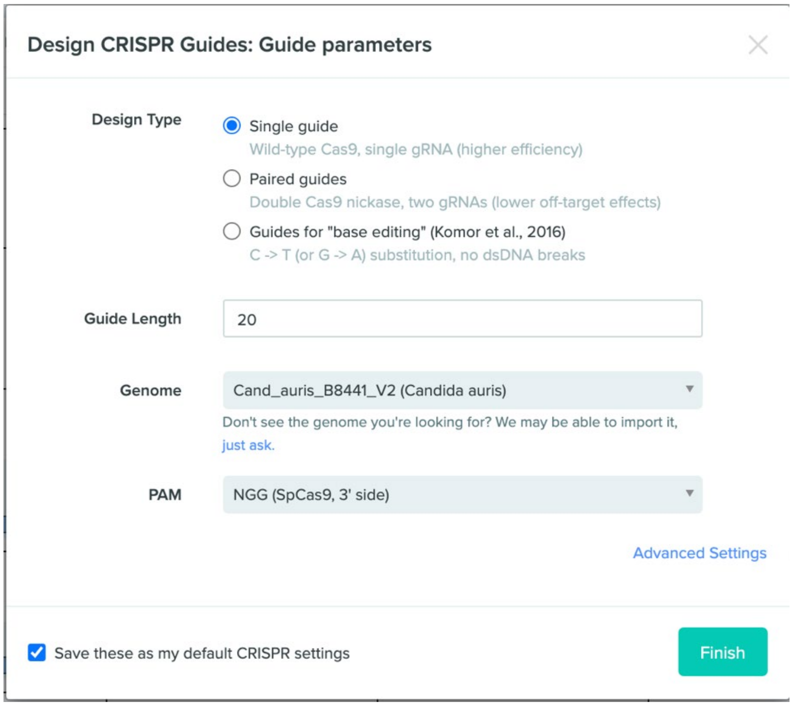

6. Click finish
7. Click the green button to confirm the target region for gRNA design

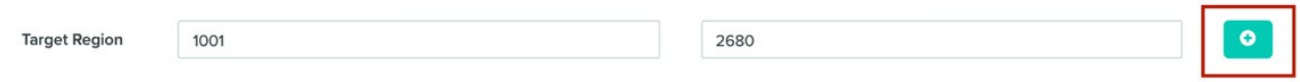

8. Sort potential gRNAs by “On-Target Score”
Note: Use the highest possible “On-Target Score” (>60+) and an “Off-Target Score” as close to 50 as possible. These values are determined by the uniqueness of the gRNA sequence and the likelihood to create off-target edits (1, 2). Remember to copy the reverse complement if the gRNA is on the – strand.

	A	B	C	D	E
1	gRNA Target	gRNA suffix	gRNA Target Sequence	Oligo Name	Target-specific gRNA oligo sequence
2	Target ORF Name	-1	[20bp target seq]		GACTCCCTCAGCAGAAAACA[20bp target seq]GTTTTAGAGCTAGAAATAGC
3	B9J08_003951 (ADE2)	pCE27	TGCCGTGAAAGAAAACACCA		GACTCCCTCAGCAGAAAACATGCCGTGAAAGAAAACACCAGTTTTAGAGCTAGAAATAGC
4					
5					
6					

10. Order the 60 bp custom gRNA oligo. This will be used for amplifying the unique B Fragment.

The repair template for markerless transformation is amplified from genomic DNA and can be made quickly and easily for multiple genes at once. This is done by replacing the open reading frame or feature of interest with a 23 bp custom AddTag for simple reconstitution of the gene to the native locus (3, 4).

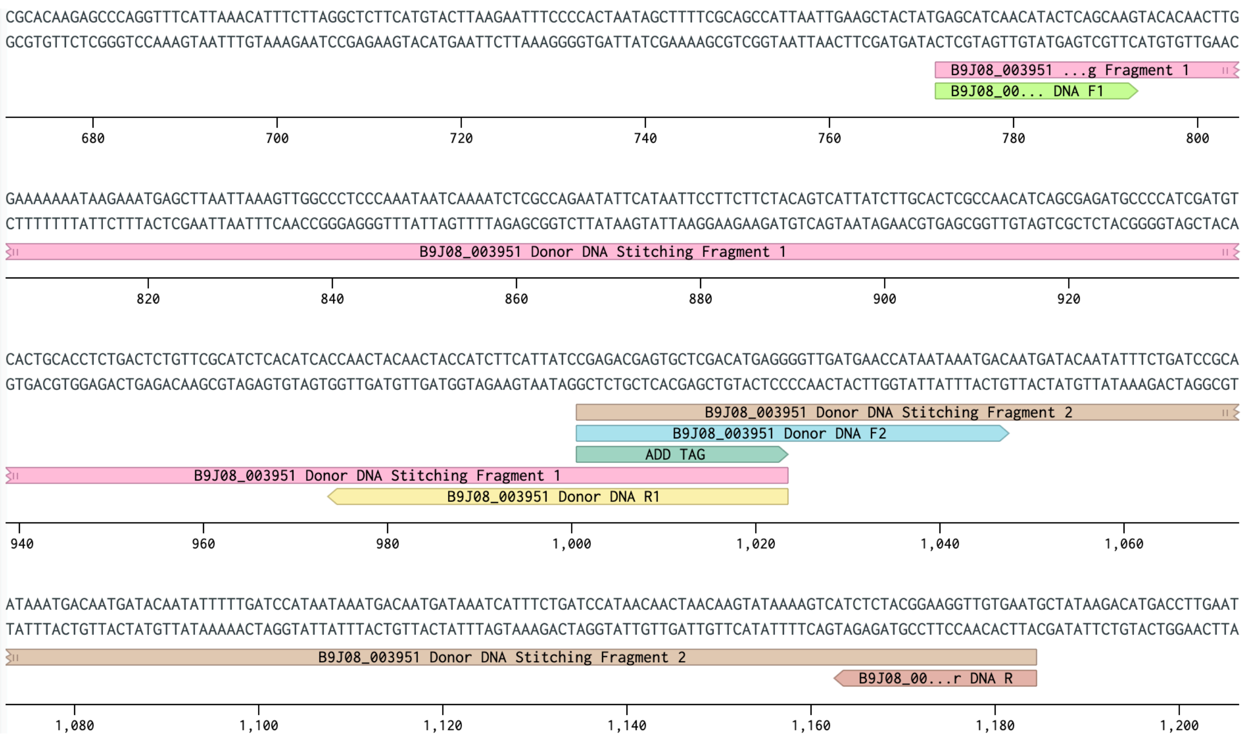

Begin by removing your gene of interest on Benchling and replacing it with the 23 bp (CGAGACGAGTGCTCGACATGAGG) AddTag. Design a forward and a reverse primer that sit 100-200 bp upstream and downstream of this newly inserted AddTag. Select primers with a T_m of approximately 60°C.

Next design a forward and reverse primer with approximately 20 bp homology to the genome with the AddTag sequence included as shown above.

Use these primers to amplify two different fragments for subsequent PCR stitching.

Donor DNA Fragment Amplification PCR Reaction	
dH_2O	37.5 µL
5x HF Buffer	10 µL
DMSO	2.5 µL
10 mM dNTPs	1 µL
Genomic DNA	100-200 ng
FWD Primer (100 µM)	0.25 µL
REV Primer (100 µM)	0.25 µL
Phusion Taq	0.25 µL

PCR Cycling Conditions

Temp (°C)	Time	Cycles	
98	30 s	1	
98	10 s	10	Δ-1 C / cycle
65	20 s		
72	15 s		
98	10 s	25	
60	20 s		
72	15 s		
72	15 s	1	
8	Hold	1	

Check 5 µL on a gel and confirm this was amplified correctly. If both fragments amplified, move forward with the stitching protocol. Adjust annealing temperature as needed for efficient amplification.

Donor DNA Stitching PCR Reaction Mix	
dH_2O	74.5 µL
5x HF Buffer	20 µL
10 mM dNTPs	2 µL
Donor DNA Fragment 1	1 µL
Donor DNA Fragment 2	1 µL
Phusion Taq	0.5 µL

First round of PCR

Temp (°C)	Time	Cycles
98	30 s	1
98	10 s	5
58	20 s	
72	15 s	

Add 0.5 µL each of the Forward 1 and Reverse 2 primers that sit 100-200 bp upstream of the start/stop codon used to amplify fragment 1 and 2 respectively. Mix well. Use the following PCR cycling conditions.

Second round of PCR

Temp (°C)	Time	Cycles
98	30 s	1
98	10 s	
66	20 s	
72	15 s	30
72	20 s	1
8	hold	1

Universal A and B Fragments

These PCR fragments are amplified from pCE27 (NAT) or pCE41 (HYG) and are used to construct the full gRNA containing C Fragment. The A fragment contains the NAT or HYG 2 of 2 marker with homology to the NAT or HYG 1 of 2 marker on the Cas9 plasmid and the *C. auris SNR52* promoter driving expression of the unique gRNA. The gRNA for amplification should be designed as described in the gRNA Design section of this protocol. The B fragment contains the conserved *tracr*RNA required for directing Cas9 and 3' homology to the *C. auris LEU2* (B9J08_000229) ORF.

A and B Fragment Reaction Mix

	A Fragment	B Fragment
dH_2O	75.5 µL	75.5 µL
5X HF Buffer	20 µL	20 µL
10 mM dNTP	2 µL	2 µL
FWD Primer (100 µM)	0.5 µL (AHO1096)	0.5 µL unique gRNA
REV Primer (100 µM)	0.5 µL (CJNO3235)	0.5 µL (AHO1097)
pCE27 or pCE41 (1 ng/µL)	1 µL	1 µL
Phusion Taq	0.5 µL	0.5 µL
Total Reaction Volume	100 µL	100 µL

A and B Fragment Cycling Conditions

Temp (°C)	Time	Cycles
98	30 s	1
98	20 s	
58	20 s	30
72	30 s	
72	15 s	1
8	∞	1

Check 5 µL of each fragment on a gel. Expected PCR amplicon for each fragment is approximately 1 kB.

C Fragment Stitching

The C Fragment is created by stitching the Universal A and Unique B Fragments together.

C Fragment PCR Reaction Mix	
dH_2O	74.5 µL
5x HF Buffer	20 µL
10 mM dNTPs	2 µL
Universal A	1 µL
Unique B	1 µL
Phusion Taq	0.5 µL

Temp (°C)	Time	Cycles
98	30 s	1
98	10 s	5
58	20 s	
72	60 s	

Add in 0.5 µL each of 100 µM of C Fragment Forward (NAT:AHO1237 or HYG:3755) and Universal C Fragment Reverse (CJNO3080). Mix well.

PCR Stitching 2 Cycling Conditions:

Temp (°C)	Time	Cycles
98	30 s	1
98	10 s	30
66	20 s	
72	60 s	
72	30 s	1
8	hold	1

Successful amplification of the C Fragment can be confirmed by checking 5 µL on a gel with an expected amplicon of 2 kB. If this stitching procedure results in poor yield, multiple bands, or smearing on the gel, consider a PCR cleanup of the A and B Fragments prior to amplification.

Cas9 Plasmid Digestion

The Cas9 construct is contained in pCE35 (NAT) or pCE38 (HYG) and is digested for use in *C. auris* using *Mss*I prior to transformation. The Cas9 plasmid has homology to the 5' end of *C. auris LEU2* (B9J08_000229) orf with the *C. auris ENO1* promoter driving expression of Cas9. Downstream of Cas9 is the NAT or HYG 1 of 2 marker with homology to NAT or HYG 2 of 2 on the gRNA plasmid.

Digest 2500 ng of pCE35 or pCE38 with *Mss*I per transformation reaction. Confirm successful digestion on a gel.

C. auris Transformation by Heat Shock

1. Streak strain(s) on a YPD plate and incubate for 2 days at 30°C
2. Inoculate 4 mL of YPD with a single colony and incubate for 12-16 h shaking at 30°C
3. Measure the OD600 of the overnight culture
4. Dilute culture to an OD600 of 0.1 in fresh YPD, you'll need 5 mL per transformation
   Example: If attempting to delete 4 genes, you will need at least 20 mL of cell culture
   Note: I recommend calculating enough YPD for n + 1 to account for checking the optical density at step 6
5. Incubate this back dilution at 30°C shaking
6. Remove flask when the OD600 is between 0.5 and 0.7
   Note: this can take 4-8 h.
7. Decant cells into a 50 mL conical tube and spin down at 4000 g for 5 min
8. Remove supernatant
9. Resuspend cells in 1 mL sterile dH_2O and transfer to a sterile 1.5 mL tube
10. Spin down cells, wash again with sterile dH_2O
11. Resuspend cells in 1/100 volume of the original starting solution in sterile dH_2O
    Note: for example, if your starting culture is 20 mL, resuspend the cell pellet in 200 μL
12. Mix your cells in a clean 1.5 mL tube with 10 μL boiled salmon sperm DNA, 50 μL PCR amplified dDNA, 50 μL C Fragment, and 2500 ng *Mss*I digested Cas9 plasmid
13. Make PLATE mix for the n + 1 transformations
    Note: the PLATE mix consists of 875 μL 50% PEG, 100 μL 10X TE, 25 μL 1 M lithium acetate
14. Add 1 mL PLATE mix to the cell/DNA mixture and mix by gently inverting
15. Place the cell/DNA/PLATE mixture in an incubator overnight at 30°C
16. The following morning, heat shock cells at 44°C in a water bath for 15 min
17. Promptly remove the 1.5 mL tube and spin down
18. Carefully remove the PLATE mixture without disturbing the cell pellet
19. Wash cell pellet twice with 1 mL YPD
20. Resuspend in 1 mL YPD and transfer to a snap top tube with 1 mL YPD
21. Recover cells at 30°C shaking for 4 h
22. Note: at this stage I place the YPD plates supplemented with NAT or HYG at 30°C to prewarm. Plating on cold plates significantly reduces editing efficiency.
23. Collect snap top tubes and spin down at 4000 rpm for 5 min
24. Remove supernatant and resuspend the cell pellet in 100 μL sterile dH_2O
25. Plate resuspended cell pellets on YPD + NAT300 or YPD + HYG600
26. Incubate plates for 2-3 days at 30°C and check for the desired edits using PCR

Colony PCR Verification

1. Aliquot 100 μL 20 mM NaOH into a the well of 96 well PCR plate for each colony you check
2. Pick a transformed colony from the YPD + NAT300 or HYG600 plate and patch onto a new plate with your selectable marker, swirl remaining colony in the 20 mM NaOH

3. Repeat for all the colonies you wish to check
4. Seal the PCR plate
5. Boil in a thermal cycler at 99°C for 15 min; this creates your lysate for the subsequent PCR reaction
6. Create a PCR reaction mix with the following components, scale up as needed

dH_2O	11.66 µL
DreamTaq Green Buffer	2.2 µL
5M Betaine	4.4 µL
$MgCl_2$	0.44 µL
10 mM dNTP	0.44 µL
DreamTaq	0.22 µL
FWD Primer (100 mM)	0.22 µL
REV Primer (100 mM)	0.22 µL
Lysate	2.2 µL

Temp (°C)	Time	Cycles
94	30 s	1
94	10 s	35
55	30 s	
72	1 min	
72	15 s	1
8	∞	1

Note: I recommend checking the Tm for your PCR primers HERE and confirming this with the recommended Tm on Benchling. An annealing temp of 55°C usually amplifies most primer pairs; however, it is worth confirming prior to starting the PCR cycling conditions.

7. Run 20 µL of each reaction on a gel and image to confirm the transformation of interest

---

LEUpOUT

Colonies that are PCR verified to have made the edit of interest should undergo the LEUpOUT process to remove the CRISPR/Cas9 components and NAT or HYG resistance markers. This is done by streaking patched colonies on the YPD + NAT300 or HYG600 plates on SC media lacking leucine. Allow this plate to grow for 48-72 h, or until colonies are large enough to work with.

Colonies on SC media lacking leucine plates should be picked with a sterile toothpick or inoculation loop and patched on a YPD plate and a YPD+NAT or HYG to confirm loss of the

CRISPR/Cas9 components. If there is minimal or no growth on the YPD + NAT300 or HYG600 plate, move forward with making a -80°C freezer stock.

Note: Best practice is to confirm the intended genome edits by PCR following the LEUpOUT procedure prior to storing a -80°C freezer stock. See the "Colony PCR Verification" section for more information on creating the reaction mix and cycling conditions.

References

1. Doench JG, Fusi N, Sullender M, Hegde M, Vaimberg EW, Donovan KF, Smith I, Tothova Z, Wilen C, Orchard R. 2016. Optimized sgRNA design to maximize activity and minimize off-target effects of CRISPR-Cas9. *Nature Biotechnology* 34:184-191.
2. Hsu PD, Scott DA, Weinstein JA, Ran FA, Konermann S, Agarwala V, Li Y, Fine EJ, Wu X, Shalem O. 2013. DNA targeting specificity of RNA-guided Cas9 nucleases. *Nature Biotechnology* 31:827-832.
3. Seher TD, Nguyen N, Ramos D, Bapat P, Nobile CJ, Sindi SS, Hernday AD. 2021. AddTag, a two-step approach with supporting software package that facilitates CRISPR/Cas-mediated precision genome editing. *G3 Genes|Genomes|Genetics* 11:jkab216.
4. Nguyen N, Quail MMF, Hernday AD. 2017. An Efficient, Rapid, and Recyclable System for CRISPR-Mediated Genome Editing in *Candida albicans*. *mSphere* 2:e00149-17.

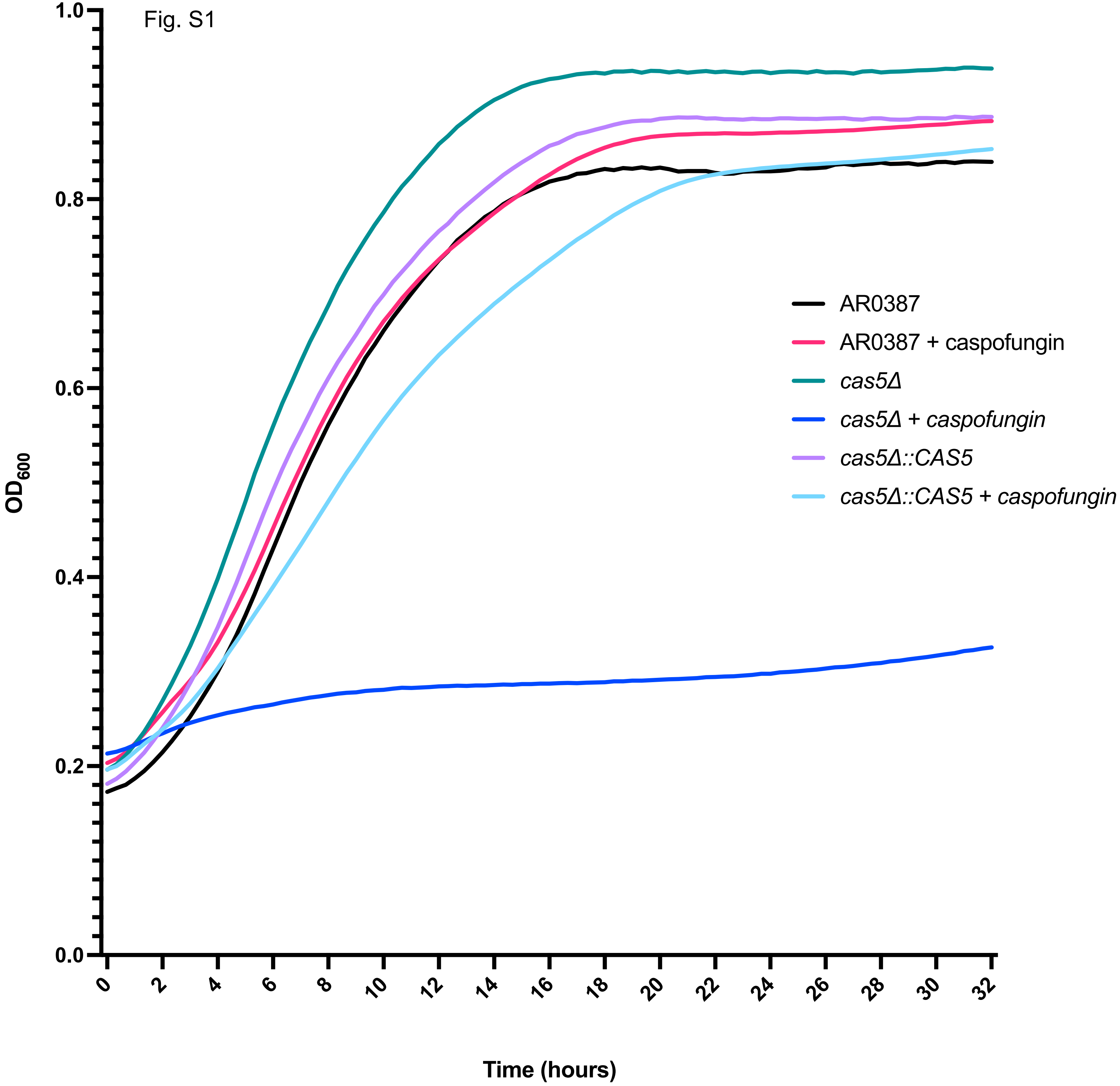
Fig. S1
OD600
Time (hours)
1.0
0.8
0.6
0.4
0.2
0.0
0
2
4
6
8
10
12
14
16
18
20
22
24
26
28
30
32
AR0387
AR0387 + caspofungin
cas5Δ
cas5Δ + caspofungin
cas5Δ::CAS5
cas5Δ::CAS5 + caspofungin

FIG S1. Growth rates of the *C. auris* wildtype, *cas5*Δ, and *cas5*Δ::*CAS5* strains. (A) Growth rates were measured for *C. auris* clade I wildtype (AR0387), *cas5*Δ (CEC99), and *cas5*Δ::*CAS5* (CEC183) strains. Strains were grown in RPMI-1640 medium alone or supplemented with 62.5 ng/mL caspofungin for 32 h with shaking at 30°C. OD_{600} readings of the individual wells were measured every 20 min. Twelve replicates per strain and growth condition were performed, with the average displayed for each strain.
